# Release of circulating tumor cells and cell-free nucleic acids is an infrequent event in synovial sarcoma: liquid biopsy analysis of 15 patients diagnosed with synovial sarcoma

**DOI:** 10.1186/s13000-018-0756-2

**Published:** 2018-10-17

**Authors:** Dóra Mihály, Noémi Nagy, Gergő Papp, Zsuzsanna Pápai, Zoltán Sápi

**Affiliations:** 1 1st Department of Pathology and Experimental Cancer Research, Semmelweis University, Üllői út 26, Budapest, H-1085 Hungary; 2 Department of Oncology, Military Hospital Budapest, Podmaniczky utca 111, Budapest, H-1062 Hungary

**Keywords:** Liquid biopsy, Synovial sarcoma, SS18-SSX fusion transcript, Droplet digital PCR, Nested PCR

## Abstract

**Background:**

Synovial sarcoma is a rare soft tissue tumor which contains the unique SS18-SSX1, SS18-SSX2 – or, rarely, SS18-SSX4 - fusion transcripts. It is well known that some soft tissue tumors, like Ewing sarcomas and myxoid liposarcomas, can spread via the blood with free circulating tumor cells (CTC); this can be detected by several sensitive molecular biology methods. Here we report a study of fifteen synovial sarcoma patients with varied clinical backgrounds.

**Method:**

After blood withdrawal and nucleic acid isolation, we attempted to detect the SS18-SSX fusion genes from circulating tumor cells or cell-free nucleic acids with nested PCR and droplet digital PCR.

**Results:**

SS18-SSX2 fusion transcript was identified in a small copy number with droplet digital PCR in one case. Nested PCR could not detect any of the fusion transcripts in the examined 15 synovial sarcoma cases.

**Conclusions:**

Heretofore two case reports could detect CTCs in synovial sarcoma - in the first paper, the patient was diagnosed with poorly differentiated type while the other had a rare primary gastric synovial sarcoma. However, until now, no other studies have detected CTCs in the peripheral blood of synovial sarcoma patients. Based on our findings, we can conclude that detection of the chimeric SS18-SSX fusion gene after surgical excision and/or chemotherapy/radiotherapy is a rare circumstance and hence in itself is not sufficient for monitoring the tumor recurrence. Therefore, monitoring of other possible biomarkers - for example synovial sarcoma specific miRNAs - is recommended.

## Background

Synovial sarcoma (SS) constitutes the third most common malignant soft-tissue tumor group and usually affects teenagers and young adults [1, 2]. Despite the combined therapeutic effort (chemotherapy and/or radiotherapy and surgical excision), the recurrence of the tumor and metastasizing ability is high [3]. The characteristic and specific translocation t(X;18((p11.2q11.2)) of human SS causes the fusion of the SS18 (also known as SYT) gene on chromosome 18 to SSX1, SSX2 or, rarely, SSX4 on chromosome X at Xp11.2, and contributes to the formation of SS18-SSX fusion transcripts, regardless of histologic subtype [4–7]. The function of the SS18-SSX chimeric protein and its relationship to tumor development remains unknown, as does the mechanism through which the translocation occurs [8]. The presence of the SS18-SSX fusion transcripts facilitates the specific and sensitive diagnosis of SS using either conventional RT-PCR, qRT-PCR or dual color FISH as diagnostic tools in fresh tumors or paraffin-embedded tissues [9]. The sensitivity of FISH and RT-PCR is more than 90% for the diagnosis of SS [10]. It seems according to a recent paper that the sensitivity of the different PCR methods (such as qPCR, nested PCR and droplet digital PCR) varies throughout different studies, being most probably dependent on the assay design and PCR product size [11].

Circulating tumor cells (CTCs) can be detected from the peripheral blood and, in theory, show the potential to extravasate and create tumor metastases. Detection of CTCs has been examined across a wide spectrum of tumor types, but most commonly in carcinomas of the breast, lung and colon, as well as in melanoma [12]. Liquid biopsy, as a less invasive and easily performable method of sample collection is nowadays a popular diagnostic tool of tumor detection and follow-up via the detection of CTCs and cell free nucleic acids, like cell free DNA (cfDNA) and cell free RNA (cfRNA) [13]. In contrast to carcinomas, few studies have investigated the identification of CTCs in sarcomas [14]. Among mesenchymal tumors, CTCs have been for the most part well-studied in Ewing’s sarcoma/peripheral neuroectodermal tumor (EWS/PNET) [15–18]. Regarding CTCs in SS, less data is available where investigators have identified the fusion gene product from the peripheral blood of affected patients. Hashimoto el al. [19] analyzed serial blood samples of a poorly differentiated SS patient before and after treatment, and CTCs with SYT-SSX fusion gene were found in the peripheral blood before, but not after, surgical resection of the primary tumor and the first cycle of chemotherapy. The in vitro study of Fricke et al. [11] revealed the SYT-SSX fusion gene transcript in both SS cells and microvesicles. However, the fusion gene transcript was not detected in peripheral blood cells and microvesicles of SS. Therefore, it is imperative to reveal the accidental genometastasis formation of SS in order to understand the tumor biology of synovial sarcomagenesis and to find new potential diagnostic markers. Thus, the aim of this study was to further examine and validate CTC detection in SS as a tool for prognostication or patient surveillance.

## Methods

### Patient selection

We collected 15 cases of SS diagnosed at Semmelweis University, Budapest, between 2009 and 2015. Beside the characteristic histology and immunophenotype of SS (cytokeratin, epithelial membrane antigen (EMA), Bcl2, TLE-1, SYT, β-catenin, vimentin, cyclin D1, CD117, SMARCB1), the translocation of SS18 and SSX1 or SSX2 fusion genes confirmed by FISH with SS18/SSX TriCheck™ Probe (ZytoVision, Bremerhaven, Germany) verified the diagnosis of SS in the 15 selected cases. Clinical data were gathered from the database of Semmelweis University, and treatment protocols were collected from the Department of Oncology at Military Hospital, Budapest, retrospectively. Signed informed consent was obtained from all participants, allowing analysis of tumor tissue, clinical data, and blood samples, and the study was conducted with the approval of the Semmelweis University Regional and Institutional Committee of Science and Research Ethics and according to the Declaration of Helsinki.

### Sample collection and RNA isolation

A 10 ml peripheral blood sample was collected in heparinized tubes from each of the patients diagnosed with SS. The clinical data of the patients can be found in Table 1. The blood withdrawal was carried out about six months after surgical treatment and/or chemotherapy or radiotherapy. At the time of blood sample collection, 7 patients had multiplex metastases, 4 had metastases, 3 had metastases and recurrences, 2 had recurrences only and in 3 cases, there was no metastasis nor recurrence; in this way, the selected cohort represented cases involved in practically all clinical stages (Table 1). Total RNA of the collected (10 ml) of peripheral blood samples was isolated with TRIzol® Reagent (Invitrogen by Thermo Fisher Scientific, Rockford, IL, USA) according to the manufacturer’s protocol. An SS18-SSX1-carrying human immortalized adipose derived mesenchymal stem-cell line (SS-iASC) [20] and two formalin-fixed paraffin-embedded (FFPE) SS tissue samples were used as positive controls. SS-iASC was cultured in 25 cm^2^ tissue culture flasks for 48–72 h, then total RNA was isolated using the PureLink RNA Mini Kit (Invitrogen by Thermo Fisher Scientific) according to the manufacturer’s protocol. As an optional step, we removed residual DNA by DNAse treatment. RNA was extracted from FFPE tissue by using RecoverAll Total Nucleic Acid Isolation Kit (Ambion by Thermo Fischer Scientific) according to the manufacturer’s recommendation. Yield and purity of isolated RNA were estimated by NanoDrop 1000 (NanoDrop Technologies, Houston, TX, USA). Purified RNA samples were stored at -80 °C until further investigation.

**Table 1:** Clinical characteristics of the synovial sarcoma cohort

Case	Site^a^	Size^b^ (cm)	Therapy	Site of metastasis	Size of recurrence^c^ (cm)	Subtype
1	right leg	5.5	SX + ChT	mpx lung, bone, CNS	no recurrence	monophasic
2	left ankle	4	SX + ChT	lung	no recurrence	biphasic
3	lung	NA	SX + ChT	lung	no recurrence	biphasic
4	gluteal region	7	SX + ChT	no metastasis	no recurrence	monophasic
5	left hand	1	SX + ChT	mpx lung	2.8	monophasic
6	left praesacral region	NA	SX + ChT	mpx lung	4	monophasic
7	left leg	NA	SX + ChT	mpx lung, liver	3	monophasic
8	abdominal wall	10	SX + ChT	no metastasis	3.5	monophasic
9	right palm	3	SX	no metastasis	1.5	biphasic
10	neck	NA	SX + RT + ChT	no metastasis	no recurrence	monophasic
11	left forearm	NA	SX + ChT	lung	2.1	monophasic
12	right forearm	3.9	SX + ChT	mpx lung, mediastinum	no recurrence	monophasic
13	right popliteal region	10	SX + ChT	mpx lung, kidney	NA	monophasic
14	right sole	5	SX + ChT	no metastasis	no recurrence	monophasic
15	left leg	NA	SX + ChT	mpx lung, pleura, mediastinum	no recurrence	biphasic

^a^location of the primary tumor; ^b^maximum diameter of the primary tumor; ^c^maximum diameter of the recurrent tumor; *NA* not available, *SX* surgical excision, *ChT* chemotherapy, *RT* radiotherapy, *mpx* multiplex, *CNS* central nervous system. Chemotherapy means the combination of the following agents: Epirubicin; VIP: 3 cycles of Etoposide, Ifosfamide and Cisplatin; Bisphosphonate; Dacarbazine; Vincristine; Pazopanib; Doxorubicin; Ifosfamide; Gemcytabine; Docetaxel; Carboplatin; Etoposide; CYVADIC: Cyclophosphamide, Vincristine, Adriamycin, Dacarbazine

### Reverse transcription

The RNA samples were reverse-transcribed using the High-Capacity cDNA Reverse Transcription Kit (Applied Biosystems, Foster City, CA, USA) to synthetize first-strand complementary DNA. Until PCR measurements were taken, the reverse transcribed cDNA samples were stored at -20 °C.

### Nested PCR and droplet digital PCR

To demonstrate the SS18-SSX1 or SS18-SSX2 fusion genes, nested PCR and droplet digital PCR (ddPCR) were accomplished. Nested PCR was carried out with two sets of primers based on the modified protocol of Hashimoto et al. [19]. PCR primers for the first-round PCR were the following: 5’-CAACAGCAAGATGCATACCA-3′ and 5′- CACTTGCTATGCACCTGATG-3′. For the second-round PCR, the following primers were used: 5′- ACAGCCTGGACCACCACAGC-3′ and 5′- AGGCATGTTTCCCCCTTTTG-3′. Denaturation for 30 cycles at 95 °C for 20 s, annealing at 58 °C for 20 s and extension at 72 °C for 20 s were performed after initial denaturation for 10 min. The primer set of the first-round PCR yielded PCR products of 548 base pairs (bp), while the primers of the second-round were designed to amplify both SS18-SSX1 and SS18-SSX2 subtypes and to form considerably shorter products with 212 bp. As positive controls, SS-iASC and two paraffin-embedded SS tissues (SS1 and SS2) were used where translocation of SS18 and SSX1 or SSX2 genes was confirmed previously by FISH. In the tissue samples and the SS-iASC, the coding region of the SS18-SSX fusion gene was analyzed solely by single PCR using the second-round primers of the nested PCR. As an internal control, GAPDH was used. The length of the PCR products was investigated with agarose gel electrophoresis. Bands were detected using the Kodak Image Station 4000MM (Kodak, Rochester, NY, USA). ddPCR was accomplished using the SS18-SSX1-positive FAM (Hs03024820_ft) and the SS18-SSX2-positive FAM (Hs03024398_ft) Taqman assays (Applied Biosystems) and the QX200 ddPCR system (Bio-Rad Laboratories, Hercules, CA, USA) according to the manufacturer’s protocol. At first, cDNA samples were divided into droplets by the QX200 Droplet Generator (Bio-Rad). Thus, PCR amplification was carried out within each droplet using the C1000 Touch™ Thermal Cycler (Bio-Rad). After PCR, droplets were flooded in a single file on a QX200 Droplet Reader (Bio-Rad) which counted the fluorescent positive and negative droplets. After counting, the analysis software QuantaSoft™ (Bio-Rad) calculated the copy number or concentration of the target RNA. Event counts < 3 were interpreted as undetected since negative controls appeared in 3 events. As negative controls, SS18-SSX non expressing cell lines named iASC [21], Caco2 and HT-1080 were used while as positive controls, the aforementioned two paraffin-embedded SS tissues and SS-iASC were used.

## Results

### Clinical and histopathological findings

**Fig. 1:**
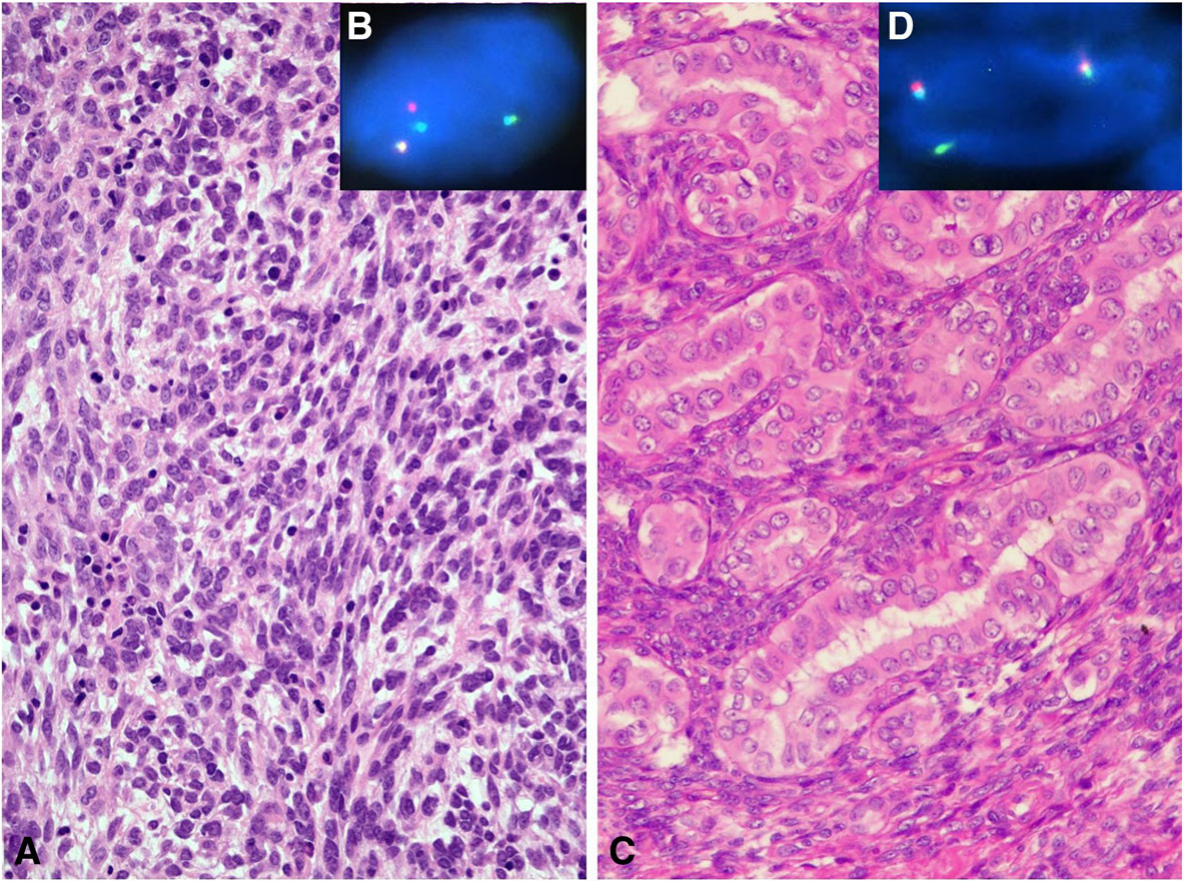
Hematoxylin-eosin (H&E) staining and FISH analysis of two SS patients. **a** H&E staining of a monophasic, spindle cell SS (*case no. 6*). **b** The *SS18-SSX2* fusion is indicated by one separate green signal, one separate orange signal and a blue signal in close proximity of the separated green signal using FISH method. **c** H&E staining of a biphasic SS (*case no. 15*). **d** The *SS18-SSX1* fusion is indicated by one separate orange signal co-localizing with one blue signal using FISH

The clinical findings are summarized in Table 1. In our series of 15 patients with SS, the male-to-female ratio was 4:11, with patients being exclusively Caucasian. The median age at diagnosis was 45 years (range: 24–72). The tumor was located on the periphery in 9 (60%) cases and centrally in 6 (40%) cases. Tumors were larger than 5 cm in 5 (33.3%) cases. At the time of diagnosis with active SS (included in the study population), 10 patients (66.7%) presented with primary metastasized disease, while 5 (33.3%) displayed with localized disease. 11 (73.3%) tumors were classified histologically as monophasic, while 4 (26.7%) were described as biphasic. Unique histology, immunophenotype and FISH with SS18/SSX TriCheck™ Probe approved the diagnosis of SS in all cases (Fig. 1). One patient was only surgically treated, treatments of the remaining 14 (93.3%) patients included surgical excision and chemotherapy and radiotherapy (RT) before blood withdrawal. Recurrence occurred in 6 (40%) cases and all recurrent tumors were smaller than 5 cm.

### Nested PCR and ddPCR

**Fig. 2:**
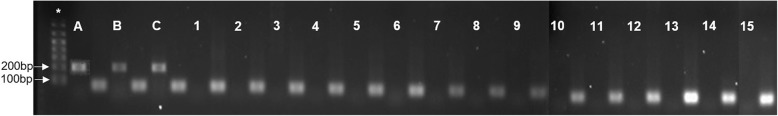
SS18-SSX nested PCR of the SS patients. Two FFPE SS tumor samples (A, B) and the SS-iASC cell line (C) were used as positive controls. Bands corresponding to the SS18-SSX fusion gene are the size of 212 bp. 1–15: cases 1–15 with no detectable SS18-SSX fusion gene. As an endogenous control GAPDH was used (92 bp). * Molecular weight marker 1 kb Plus DNA Ladder (Life Technologies for Thermo Fisher Scientific)

**Fig. 3:**
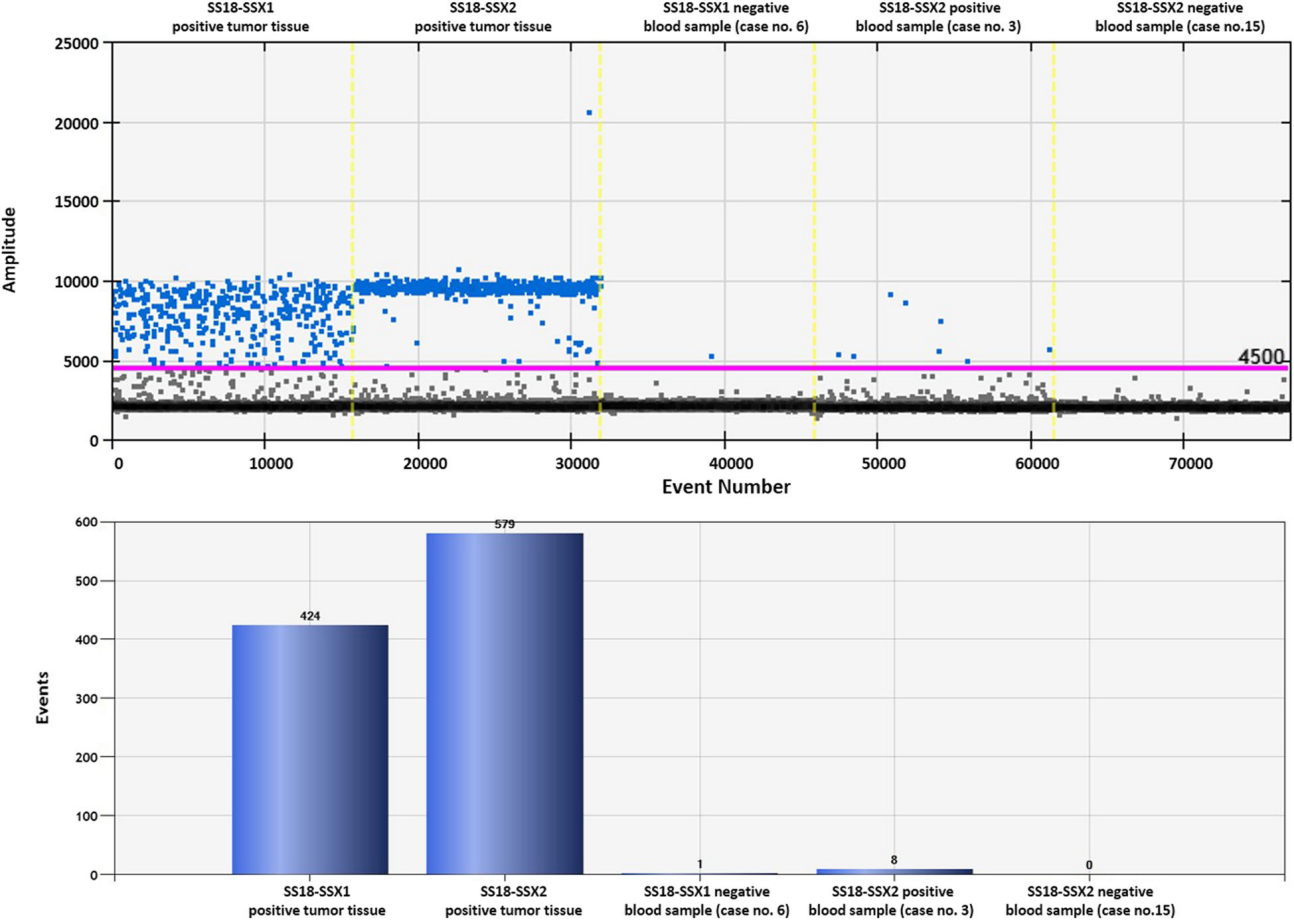
Result of the droplet digital PCR. The upper panel represents one-dimensional plot of droplets measured for a fluorescence signal emitting from the SS18-SSX1 or SS18-SSX2 positive sample (amplitude is indicated on a vertical axis, while the number of events is indicated on the horizontal axis). Positive droplets are shown in blue. Negative droplets are shown in grey. The lower panel shows the event numbers in column charts of two FFPE tumor samples used as positive controls and three blood samples (case 6, 3 and 15). In case no. 3, we could detect eight events representing the SS18-SSX2 fusion transcript as processed by QuantaSoft™ (Bio-Rad). Cases no. 6 and 15 were negative to the SS18-SSX1 and SS18-SSX2 fusion genes

Results of the agarose gel electrophoresis after nested PCR are shown in Fig. 2. The 212-bp band that represents the SS18-SSX fusion gene was only observed in the positive control SS-iASC and the two SS tissue samples while it was not detected in any peripheral blood samples of the 15 SS patients. The ddPCR results can be seen in Fig. 3 and Table 2. Regarding the positive control samples, 424 events specific for SS18-SSX1 transcript were counted in SS1, 579 events specific for SS18-SSX2 transcript were counted in SS2 sample and 51 SS18-SSX1-positive events were measured in SS-iASC (Table 2). As Fig. 3 represents, we could detect 8 SS18-SSX2-positive events in one liquid biopsy sample using the ddPCR method. The remaining 14 samples were considered as negative, since three or less events were detected with both SS18-SSX1 and SS18-SSX2 assays.

**Table 2:** Results of the ddPCR

Case no.	Event Number (SS18-SSX1)	Event Number (SS18-SSX2)
SS1	424	0
SS2	0	579
SS-iASC	51	0
1	0	1
2	0	3
3	1	8
4	0	2
5	0	2
6	1	1
7	0	1
8	1	3
9	0	2
10	0	3
11	2	1
12	2	3
13	0	1
14	1	1
15	0	0
iASC	0	3
Caco2	1	1
HT-1080	2	2

SS1-SS2: paraffin-embedded synovial sarcoma tumor tissue no. 1 and 2, SS-iASC: SS18-SSX1 expressing immortalized adipose tissue-derived mesenchymal stem cell line, iASC: immortalized adipose tissue-derived mesenchymal stem cell line, Caco2: human colon adenocarcinoma cell line, HT-1080 human fibrosarcoma cell line

## Discussion

To date, the detection of CTCs is an expansive field in tumor research and diagnostics. In lung cancer, detection of CTCs refers to the presence of metastases and the actual stage of disease [22]. In the last few years, several research groups inspected fusion transcripts derived from CTCs and cell free nucleic acids in the blood stream of EWS patients [23, 24]. The recurrent abnormal gene fusion product can be detected in involved patients but not in healthy individuals [14]. Sarcoma-specific fusion gene transcripts like EWSR1-ERG or EWSR1-FLI1 in EWS or PAX3-FKHR or PAX7-FKHR in alveolar rhabdomyosarcoma (aRMS) are providing a promising way to detect CTCs [25, 26]. Nevertheless, except for two case reports [19, 27], no further studies have validated CTCs in SS as a prognostication tool. For these reasons, we aimed to investigate the potential attendance of chimeric SS18-SSX fusion transcripts in the blood samples of SS patients after about six months of first combined therapy including surgical excision and chemotherapy and/or radiotherapy.

As mentioned above, two research groups [19, 27] could detect the SS specific SS18-SSX fusion transcript with nested PCR in the peripheral blood of two patients before but not after combined treatments. Hashimoto et al. explained the release of CTCs into the blood with the poorly differentiated histologic type of SS. Despite subsequent metastases, they could not detect the fusion gene after surgical excision and chemotherapy [19]. Correspondingly, Ogino et al. detected the unique fusion transcript preoperatively in the plasma of a patient with gastric SS but they were unable to reveal it six months postoperatively [27]. Both groups supposed that the amount of CTCs and microvesicles in these two SS patients decreased to an imponderable level.

Several SS samples were examined by Fricke et al., including the well-known 1273/99 SS cell line and blood samples of eight SS patients, and they found that RT-qPCR and nested PCR were more sensitive in the detection of SS18-SSX fusion transcripts in contrast to ddPCR. They could only detect the SS18-SSX2 fusion gene transcripts in the positive control 1273/99 cell line, but not in the liquid biopsies of the investigated patients [11]. Along with Fricke et al., we also performed nested PCR and the relatively new and pioneering ddPCR method to analyze the collected liquid biopsy samples. The whole peripheral blood of fifteen SS patients with diversified clinical backgrounds referring to their age, stage of the tumor and treatment strategies were examined. The SS18/SSX2 fusion transcript was detected by ddPCR in one patient representing the transcript in a small amount (8 positive events) of copy number (Fig. 3). Although this patient (case no. 3) had no recurrence, but lung metastasis was present at the time of blood collection. SS patients are usually recalled for detailed clinical re-staging examination (physical and radiological examination, laboratory testing) after approximately 6 months of the initial diagnosis. This approximately half-year period was chosen to try to detect the specific fusion transcript using liquid biopsies. In 12 cases there were recurrences and/or metastases, but in 3 cases there were no detectable tumor, however in the latter cases we still wanted to be sure to rule out the possibility of circulating tumor cells. A recent study underlines our findings because digital PCR-based methods, like ddPCR, have the potential to improve the limitations of RT-qPCR as Mehle et al. describe in their article. They found that reverse transcription droplet digital PCR (RT-ddPCR) as an absolute quantification method was 10-fold greater in sensitivity than RT-qPCR [28]. Pilot studies of different translocation-positive sarcomas showed that the most promising in CTC detection is the detection of the unique fusion transcript of the disease. Albeit this statement was reinforced mainly by studies of CTCs in EWS patients [14]. Recently, Uotani et al. found a novel circulating microRNA miR-92b-3p as a promising new biomarker of SS monitoring [29]. They examined the plasma from SS patients and cell culture media from SS cell lines to identify novel microRNAs. MiR-92b-3p seemed to be significantly higher in SS patients compared to healthy individuals and correlated with tumor dynamics in animal experiments.

Summing up, based on our and others’ [11, 19, 27] findings, additional SS specific biomarkers are needed to complete the detection of disease recurrence in liquid biopsies of the tumor. Moreover, it would be important to clear up whether the presence of CTCs or cell-free nucleic acids in SS correlates with the prognosis of the disease. This theory is yet to be proven.

## Conclusion

In conclusion, we can declare that the release of SS CTCs or cell-free nucleic acids is a rare occurrence and, because of this, is not a reliable marker for detecting tumor recurrence. Therefore, to diagnose minimal residual disease, predict disease recurrence or metastases before clinical manifestations and to be able to continuously monitor treatment effects, other new potential biomarkers are needed beside the detection of SS fusion transcripts.

## Abbreviations

aRMS: alveolar rhabdomyosarcoma
cfDNA: cell free DNA
cfRNA: cell free RNA
CTC: Circulating tumor cell
ddPCR: droplet digital PCR
EWS/PNET: Ewing’s sarcoma/peripheral neuroectodermal tumor
FISH: Fluorescent in situ hybridization
GAPDH: Glyceraldehyde-3-phosphate dehydrogenase
miRNA: micro-ribonucleic acid
PCR: Polymerase chain reaction
qRT-PCR: Quantitative real-time PCR
RT: Radiotherapy
SS: Synovial sarcoma
SS-iASC: SS18-SSX1-carrying human immortalized adipose derived mesenchymal stem-cell line
